# Is Smaller Better? A Proposal to Use Bacteria For Neuroscientific Modeling

**DOI:** 10.3389/fncom.2018.00007

**Published:** 2018-02-23

**Authors:** Archana Ram, Andrew W. Lo

**Affiliations:** ^1^Department of Electrical Engineering and Computer Science, Massachusetts Institute of Technology, Cambridge, MA, United States; ^2^Sloan School of Management, Massachusetts Institute of Technology, Cambridge, MA, United States; ^3^Laboratory for Financial Engineering, Massachusetts Institute of Technology, Cambridge, MA, United States; ^4^Computer Science and Artificial Intelligence Laboratory, Massachusetts Institute of Technology, Cambridge, MA, United States

**Keywords:** quorum sensing, neural networks (computer), *Bacillus subtilis*, cell-cell communication, network models

## Abstract

Bacteria are easily characterizable model organisms with an impressively complicated set of abilities. Among them is quorum sensing, a cell-cell signaling system that may have a common evolutionary origin with eukaryotic cell-cell signaling. The two systems are behaviorally similar, but quorum sensing in bacteria is more easily studied in depth than cell-cell signaling in eukaryotes. Because of this comparative ease of study, bacterial dynamics are also more suited to direct interpretation than eukaryotic dynamics, e.g., those of the neuron. Here we review literature on neuron-like qualities of bacterial colonies and biofilms, including ion-based and hormonal signaling, and a phenomenon similar to the graded action potential. This suggests that bacteria could be used to help create more accurate and detailed biological models in neuroscientific research. More speculatively, bacterial systems may be considered an analog for neurons in biologically based computational research, allowing models to better harness the tremendous ability of biological organisms to process information and make decisions.

## Introduction

The number of bacteria on Earth is staggering. Conservative estimates claim that there are nearly half a million bacterial species in just 30 g of soil (Dykhuizen, 1998). Nevertheless, the myth persists that bacteria are simple organisms. The complexity of bacterial function in many ways mirrors that of eukaryotic cells: bacteria are capable of cell-cell interactions, rudimentary cellular differentiation, colonization, and even predator-prey interactions between species (Shapiro, 1988, 1998). In this review, we examine the literature demonstrating that bacterial cells, colonies, and biofilms exhibit similarities to neurons and neuronal networks, including graded action potential-like behavior, ion-based signaling, and chemical signaling.

A natural first question is “Why would one consider using bacteria instead of neurons?” First, bacteria are genetically simpler than eukaryotes. For example, the genomes of two well-studied model bacteria, *Escherichia coli (E. coli)* and *Bacillus subtilis (B. subtilis)*, are both slightly over 4Mbp long, coding for roughly 4,000 protein-coding genes (Harwood and Wipat, 2013). Compare this with the human genome, which is roughly 3Gbp long and codes for roughly 100,000 proteins. Even *C. elegans*, one of the simplest model organisms in neuroscience, has a genome size of 100 Mbp (25 times the size of a bacterial genome) and codes for almost 22,000 proteins (Fraser et al., 2000). The comparative genetic simplicity of bacteria makes them inherently an easier organism to study. Simply put, they are capable of less and, as a result, there is less to understand about their functionality.

Second, bacteria are easier to work with in a laboratory environment. One *E. coli* cell divides roughly every 20–60 minutes and colonies can be grown overnight. Combined with the fact that bacteria are able to incorporate extracellular DNA into their genome, this allows for easier and faster genetic experimentation than their eukaryotic counterparts (Cooper, 2000). Neurons, on the other hand, are terminally differentiated cells. As a result, division is much slower (Hobert, 2011).

Finally, each bacterium is an organism in itself. Consider the phenomenon of bacterial chemotaxis, which can easily be investigated in a laboratory setting (Berg and Brown, 1972). The movement of individual organisms toward chemical attractants allows for complex behavioral experimentation on single cells that may help to elucidate comparable functionality in multicellular organisms. Such well-characterized cause-and-effect behavior cannot easily be replicated in neuronal cell cultures.

We do not propose the use of bacteria as model organisms purely for the sake of behavioral research, however, but rather as a means for creating more easily analyzed biological and biologically inspired models. To create a useful biologically inspired/biological model, one should first possess a thorough understanding of the biological underpinnings of the subject. This understanding cannot be said to exist yet for the mammalian brain, or even for the less complicated nervous system of *Drosophila melanogaster*, for example, owing to the difficulty of interpreting the mechanistic basis of observed behaviors, e.g., how neuronal firing patterns lead to an action such as object recognition.

The complexity of neuronal function has hindered the rate of progress in biologically inspired modeling. For example, despite the general lack of understanding of neuronal network function, artificial neural networks (ANNs) still use as their basic computational unit a binary input/output node that is meant to be an abstraction of a neuron (Lippmann, 1987). However, this is an oversimplified view of actual neuronal function, one that prevents the network from being able to fully harness the abilities of biological neuronal networks in order to aid computation (Staelin and Staelin, 2011).

In this review, we begin with an overview of similarities between mammals and lower-order organisms, and proceed to a discussion of the ways in which bacterial communities mirror neuronal circuits and networks. By focusing on these similarities, we hope to motivate improvements in models of higher-level organisms such as mammals and to influence biologically inspired computational efforts such as ANNs.

## Similarities between bacteria and higher organisms

Hunger and satiety detection is a relatively well-conserved system among many different species, hence it is a good starting point for a discussion about inter-species similarities (Jobst et al., 2004; Bouret and Simerly, 2006; Tessmar-Raible et al., 2007; Krashes et al., 2009; Stuber and Wise, 2016). Despite hundreds of millions of years of evolutionary divergence, there are still numerous structural and molecular similarities between *Drosophila melanogaster* and mammals, which allow *Drosophila* to serve as a more easily studied proxy for the mammalian brain in this context (Bouret et al., 2004; de Velasco et al., 2007; Olsen and Wilson, 2008; Krashes et al., 2011; Alhadeff et al., 2014; Pool et al., 2014; Denis et al., 2015; Dietrich et al., 2015; Stuber and Wise, 2016). These similarities are also seen between *Drosophila* and lower-level organisms. When hungry, both bacteria and *Drosophila* larvae ascend nutrient gradients in a process known as chemotaxis. *Drosophila* larvae approach a source of nutrients in stages: first, they approach the source; then, once near the source, they reach it and overshoot it; and finally they return to the source. This motion consists of “runs” and “turns.” The “runs” predominate while the turns are abrupt, generally occurring when a decreasing chemical concentration is sensed during forward motion and the organism must change direction in order to ascend the chemical gradient. This sort of motion is akin to a biased random walk. The organism meanders toward the center of the nutrient concentration, but may wander slightly along the way (Bargmann and Horvitz, 1991; Gomez-Marin et al., 2011). The mechanism employed by *E. coli* also favors crawls toward higher nutrient concentrations rather than lower ones, and does so in a way that also employs runs and turns (Berg and Brown, 1972).

The similarities between *Drosophila* and bacteria go further than feeding behaviors. Consider quorum sensing, a form of bacterial cell-cell communication generally used to sense local bacterial population density. The protein AarA of the Gram-negative soil bacterium *Providencia stuartii* is necessary to release the molecular signals for quorum sensing in that species. This protein appears to be homologous to the *Drosophila* protease RHO, which is required to activate epidermal growth factor receptor ligands in the fly and is essential to ensuring proper wing vein development and eye organization. Indeed, RHO and AarA are so similar chemically that expressing *Drosophila* RHO in *P. stuartii* acts as a substitute for AarA expression and mutants possess relatively normal quorum sensing capabilities. Similarly, expressing *P. stuartii* AarA in *Drosophila* RHO mutants allows wing development to proceed normally, again allowing the substitution of the two homologs, despite their origin in two very different species (Waters and Bassler, 2005).

This homology is not an isolated incident. Many signaling mechanisms appear to be shared by prokaryotes and eukaryotes. In fact, the evolution of cell-cell signaling is hypothesized to have been more reliant on horizontal gene transfer from bacteria to animals than purely vertical inheritance (Hughes and Sperandio, 2008). An interesting example of this process is the enzyme glutamate decarboxylase, which catalyzes the amino acid glutamate to form the neurotransmitter GABA. This enzyme is coded by a gene acquired by eukaryotes from prokaryotes through horizontal gene transfer (Waters and Bassler, 2005).

## Similarities in bacterial and neuron ion-based communication

Bacteria have not only influenced the development of eukaryotic cell-cell signaling, but they also possess a number of direct similarities to neurons, specifically with respect to the cell membrane and their means of cell-cell communication.

Neuronal membrane voltage is regulated by the common but important ions Na^+^ (sodium), Cl^−^ (chlorine), Ca^2+^ (calcium), and K^+^ (potassium). K^+^ tends to accumulate inside the membrane, while Na^+^, Cl^−^, and Ca^2+^ have higher concentrations outside the membrane. Notably, K^+^ strongly influences the membrane voltage and is a major determinant of the resting membrane potential (Bear et al., 2007). There is a growing body of evidence that bacteria also use these ions to regulate voltage across the bacterial cell membrane. As in neurons, Na^+^ accumulates on the outside of the bacterial cell membrane, while K^+^ accumulates inside. There is even some evidence for ionic Na^+^/K^+^ exchange across the bacterial cell membrane, perhaps mediated by pumps similar to the ones found in neurons (Lanyi, 1979).

It should be noted that the structure of the K^+^ channel—which is crucial in regulating the membrane voltage in both bacteria and neurons—was first determined from a bacterial source due to the ease of bacterial study compared to that of neurons (Yellen, 1999). Additionally, the resting membrane potential of *E. coli* is −75 mV, only about 5mV lower than that of neurons, suggesting additional electrophysiological parallels between the two cell types (Schuldiner and Kaback, 1975).

**Figure 1:**
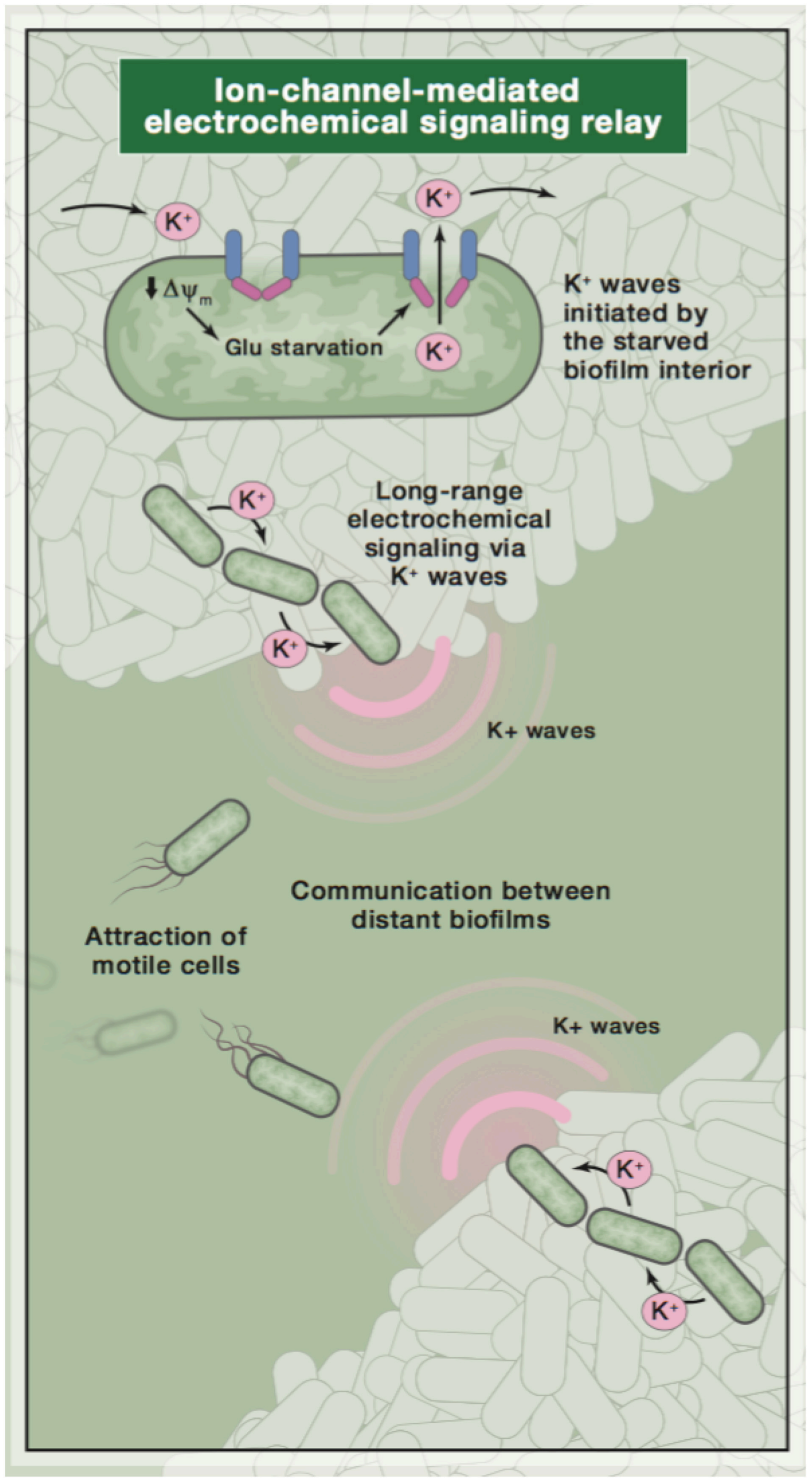
A diagram of ion-based communication in biofilms. Image reused with permission from original publisher (Humphries et al., 2017).

The effects of bacterial ionic membrane voltage regulation are clearly seen in *B. subtilis*, a Gram-positive spore-forming bacterium (Aguilar et al., 2007). Bacteria tend to produce biofilms when they are stressed, e.g., when there are limited nutrients in the environment (Prindle et al., 2015). When this species produces biofilms of greater than 1 million cells, the colony naturally produces electrical oscillations that serve to modulate the biofilm's voltage as a whole. Intracellular and extracellular potassium ions produce a gradient on the given substrate, toward which motile bacteria of various species are attracted, based on the K^+^ ion's capability to alter the resting membrane potential. This K^+^-based attraction appears to be coupled to the biofilm's oscillations, thereby producing a phenomenon similar to the graded action potential behavior seen in higher organisms (Figure 1; Humphries et al., 2017).

These commonly found ions are used in bacteria not only to regulate membrane voltage, but also as signals. The PhoP/PhoQ system in *Salmonella typhimurium* governs various virulence properties of that organism and has distinct binding sites for both Ca^2+^ and Mg^2+^. These extracellular ions act as the signals to activate the system in a way that appears analogous to the neuronal calcium channel regulator CaBP1, which also has binding sites for both Ca^2+^ and Mg^2+^ and is implicated in the facilitation of neurotransmitter release (Véscovi et al., 1996, 1997; Wingard et al., 2005; Leal et al., 2012).

## Similarities between bacterial quorum sensing and neuronal communication

Ions, however, are not the only extracellular signals present in bacteria. Similar to the neuronal use of neurotransmitters, bacteria also use complex signaling compounds to communicate. Bacterial quorum sensing is achieved through the use of different peptides and hormones, and in many ways, the process mimics how neurons communicate at chemical synapses.

There are several types of quorum sensing systems, each classified by signaling molecule. One system involves hormone-like compounds known as autoinducers (AIs), found in Gram-negative bacteria. The amino acid derivative AHL (*N*-acyl homoserine lactone) is a frequently used and studied class of autoinducer compound. AHLs are mainly used for intra-species communication between Gram-negative bacteria, useful in environments where different bacterial species share resources. The most common autoinducer used in Gram-negative bacteria, mainly used for communication within a given colony, is AI-2. This relatively universal communication molecule is used in over 40 species of bacteria. A chemically divergent type of quorum sensing system is found in Gram-positive bacteria. Rather than using autoinducers, these bacteria use modified oligopeptides as signaling molecules, which is similar to the neuronal use of peptide neurotransmitters (Snyder and Innis, 1979; Bassler, 2002).

The canonical example of quorum sensing is the system used by *Vibrio fischeri*, a bacterium that lives inside the light organ of the squid *Euprymna scolopes*. Once these bacteria grow to a high enough cell density, quorum sensing induces the expression of genes necessary for bioluminescence (Waters and Bassler, 2005). At its most basic, quorum sensing molecules passively diffuse through the bacterial membrane, accumulating both intra- and extracellularly at a concentration proportional to cell density. Once the signal has reached an appropriate level inside the cell, the transcription of certain genes will begin. In addition, this signal may also be detected through receptors. Gram-negative/AI2-based systems tend to use cytoplasmic receptors but, interestingly, Gram-positive bacteria that exhibit neuron-like oscillatory electrical communication like *B. subtilis* also possess membrane receptors similar to those in neurons (Ng and Bassler, 2009).

The ways in which quorum sensing occurs also mirror eukaryotic communication. Quorum sensing can induce the transient expression of genes and can be used by one bacterial colony to “eavesdrop” on other populations. Quorum sensing can occur in series and in parallel, and even hierarchical quorum sensing circuits exist. Sometimes quorum sensing will produce inhibitory signals within a colony, while in other cases, bacterial populations will “sabotage” a quorum sensing signal from another colony, degrading it in a process known as quorum quenching. *B. subtilis*, for example, produces an enzyme called AiiA that is capable of hydrolyzing the AHL of another soil bacterium, thereby inhibiting its external signaling attempts (Bassler, 2002; Waters and Bassler, 2005).

There are important and striking similarities between quorum sensing and neuronal communication. As mentioned above, both quorum sensing and neuronal circuits operate in series and in parallel, and both are able to develop into a hierarchical multi-circuit system (Krashes et al., 2009). Neurons can provide both excitatory and inhibitory signals, just as quorum sensing does, and neurons can communicate with each other as well as other cell types, just as quorum sensing can function between bacteria of the same species or between different species (Siegelbaum and Hudspeth, 2000; Bear et al., 2007). These parallels are unmistakable; at minimum, it is easily confirmed that abstract similarities exist between bacterial colony and neuronal function.

## Modeled bacterial systems are similar to neuronal analogs

Perhaps the most compelling evidence supporting the analogy between quorum sensing and neuronal communication, especially from a modeling perspective, comes from the decision to be competent in *B. subtilis*. Competence is a physiological state in bacteria in which a cell is capable of taking up extracellular DNA from its environment. If *B. subtilis* is under stress, concentrations of signaling molecules in the cell will rise and, in roughly 10% of cells, competence will ensue. This choice can be modeled as an analogy to the problem from physics of a particle escaping a potential barrier under the effect of noise (Schultz et al., 2009). If the cell “decides” to be competent, it has effectively escaped the barrier. Exactly the same approach has been employed to model spontaneous action potential generation (due to ion channel fluctuations) in neurons (Chow and White, 1996).

This suggests a way in which techniques and insights from modeling bacteria can influence modeling in neuroscience. Competence in *B. subtilis* is a relatively well-characterized system. Since experimentation on bacteria is easier to conduct than experimentation on eukaryotes, it is less difficult to create a robust model of this choice based on *in vitro* experimentation than it would be for a eukaryotic analog. The insights gained from investigating this system, because it is based on the same concept as spontaneous action potential generation (namely barrier escape), can then be used to create more sophisticated models of spontaneous action potential generation. This is only one example of the powerful utility of bacteria in the context of neuroscientific modeling.

## Models of quorum sensing can bolster models of neurological disorders

A discussion of the similarities between bacteria and neurons would not be complete without considering the interaction between these different systems. Not only can studying bacterial systems provide useful analogies to neuroscientific systems, but doing so can directly improve our understanding of neuroscience.

There is a known link between microbiota composition and central nervous system (CNS) disorders. In humans and other mammals, the microbiota forms a long-term symbiosis with its host. In this symbiotic relationship, it receives regulatory signals from the CNS and similarly, it is also capable of modulating CNS function. *Lactobacillus* and *Bifidobacterium* species, for example, have been shown to alleviate depression in rat models based on apparent CNS neurotransmitter regulation. *Lactobacillus* is also capable of reducing anxiety and stress (Wang and Kasper, 2014).

Perhaps the most interesting example of bacterial-neuronal interaction is late-onset autism. In these cases, the onset of autism often follows antimicrobial therapy, and the disease itself is often associated with gastrointestinal symptoms. Additionally, oral antibiotics such as vancomycin are capable of ameliorating its symptoms to some extent. It has also been shown that children with autism appear to have a greater amount of *Clostridium* and *Rumincoccus* in their stools than controls, suggesting a role for these species in the disease (Finegold et al., 2002).

Since these bacteria-CNS interactions necessarily involve signaling, it is natural to implicate quorum sensing. In fact, the quorum sensing peptide PhrCACET1 produced by *Clostridium acetoburylicum* is capable of crossing the blood-brain barrier, corroborating the association of *Clostridium* with neurological disorders, specifically autism (Wynendaele et al., 2015).

These results demonstrate that bacteria and neurons are not merely similar, but in fact are capable of interactions that appear to have profound neurological effects. Models of bacterial function not only can be used to aid the creation of models of neuronal function, they can be integrated into these models directly in order to strengthen them. For example, a model of autism may be made more representative of its underlying etiology by including a quorum sensing component. Studies of bacteria are capable of indirectly influencing neuroscientific models and have the potential for direct impact as well.

## Conclusion

Because bacteria are simpler organisms than neurons and because quorum sensing is not as sophisticated as neuronal cell-cell signaling, there are clear limitations to the amount that can be learned from bacteria and used to guide neuroscience work. However, there are still many insights that bacterial systems are capable of yielding for modeling in neuronal systems.

Consider the weighted binary input/output units that form the basis of ANNs (Lippmann, 1987; Staelin and Staelin, 2011). These units are not only an oversimplification of actual neuronal function but also incorrectly represent neuronal activity. Because of this limitation, ANNs are not able to truly use biology to their advantage to emulate the capabilities of neuronal networks and, by extension, the sophisticated brains of higher organisms (e.g., mammals).

Now, consider the bacterium. While it cannot be abstracted to a weighted binary input/output unit, its abilities can be more easily grasped by the biologist than the neuron. Like the neuron, the bacterium exhibits clear ion channel-based communication, while bacterial quorum sensing is similar to neuronal communication. Additionally, when bacteria coalesce into a biofilm, they are able to produce something akin to the graded action potential activity seen in *C. elegans*, the simplest multicellular organism on which neuroscientific research has focused over the years (Lockery and Goodman, 2009). Because of these similarities, we expect insights gained from bacterial modeling to be able to influence models of neuronal function. It should be emphasized that understanding bacteria will not necessarily allow us to better understand neurons or neuronal function directly except, perhaps, in the case of neurological disorders. We believe that bacterial research will indirectly and perhaps even directly influence neuroscience as a whole in potentially impactful ways.

Also, bacteria are by no means the “perfect” organism to influence neuroscientific research—after all, they lack neurons—but they represent a compromise. They exhibit network-like activity, and as a colony, they are capable of making decisions in ways similar to primitive multicellular organisms (Ben-Jacob et al., 2014). Their beauty comes in their simplicity and ease of study relative to other organisms. For a model to have a sound biological basis, the biology upon which it is based must be well understood. It is intuitive enough to say that the simpler the organism, the easier it is to understand it. Following this logic to its conclusion, bacteria—more specifically, bacterial colonies—are a possible stepping stone for more biologically faithful neuroscientific modeling. They have the potential to lead the way to modeling more complex organisms, especially after dedicated behavioral, molecular, and cellular work better elucidates the neuron-like aspects of their behavior.

The use of modeled bacteria has the potential to go far beyond neuroscience. There are also numerous possible benefits to computer science. An example is the ability to perform one-shot learning in humans (Lake et al., 2014). The mechanisms behind such learning are not understood well enough to produce a computational translation of this phenomenon (ignoring recent Bayesian work in this area as it attempts to “reverse-engineer the brain” instead of drawing inspiration from biology) and ANNs are not able to perform true one-shot learning (Lake et al., 2011; Salakhutdinov et al., 2012). In general, the neuronal networks within the brain from which ANNs draw inspiration are too complicated and poorly understood to allow interesting computational translation of biological phenomena such as one-shot learning into these networks.

It cannot be claimed definitively that understanding a “simple” system such as a bacterial colony will make it easier to understand neuronal network dynamics. From a computational standpoint, however, understanding a simpler system would allow us to more easily create quantitative translations of biological phenomena. Similarly, while it cannot be claimed that understanding bacteria would allow us to create a system that is capable of one-shot learning, we may be able to examine the relationship between the accumulation of quorum sensing molecules in the cell and the resultant gene expression and emergent cell behavior to better influence the choice of activation function in a given ANN. The choice of activation function can strongly influence the rate at which the system “learns,” which in turn may be a step toward a modeled system that can perform one-shot learning.

Finally, why should we bother with improving biologically inspired models at all? Are the models we have in use today—Bayesian networks, ANNs, etc.—not enough for our needs? The answer becomes obvious if we rephrase the question: Is it worth it to allow the insights gained from biological work to influence computational work? It does not go without saying that the mammalian brain is an incredibly compact, tremendously powerful organ whose capabilities generally greatly surpass the most powerful models and computers. It would be foolish not to work toward a time when the mammalian brain is not only understandable, but influential in the creation of both software and hardware. Modeling bacteria is a first step to making this possibility a reality.

## Author contributions

AR primarily wrote this paper. AWL provided guidance and helped edit.

### Conflict of interest statement

The authors declare that the research was conducted in the absence of any commercial or financial relationships that could be construed as a potential conflict of interest.
